# Mr. Bliss's Observations on the Kilburn Water

**Published:** 1801-10

**Authors:** John Bliss

**Affiliations:** Hampstead


### *To the Editors of the Medical and Physical Journal.*

GENTLEMEN,

THE analyfes which appeared in the Sixth Volume of the London Medical Review,* of the medicinal waters in this parifh, were not offered as perfectly correct, but were merely intended to be more fo than any hitherto publifhed.

Indeed it would have been prefumption, even in the prefent advanced ftate of chemical fcience, to contend that the exact proportions of fubftances which enter into the compofition of a clafs of bodies, like that of mineral waters, could be afcertained with abfolute precifion and minutenefs.

I am led into thefe obfervations in confequence of fome animadverfions on the analyfis of the *Kilburn water*, which appeared in your Journal of laft month; and which would have been unnoticed by me, had they not contained a pofition tending to miflead thofe of your readers who are not thoroughly acquainted with the refpective affinities of bodies in their various combinations.

The ftyle and manner of this criticifm I fhall pafs over; it is the matter only which deferves my attention.

The

---

* I have, fince that period, fubmitted my papers to the infpection of an accurate chemift, with the intention of republifhing them in a feparate tract, which I fhall take an early opportunity of doing.

The gentleman who has taken the trouble to criticife my analyfis, has no doubt, from the nature of his late employment, obtained *some* chemical information. Is it not to be regretted, that he had not acquired a more extenfive knowledge of the fcience, and a more perfect acquaintance with this branch of it, before he afcended the critical chair?

Two circumftances only appear to have called for his animadverfions, viz. the ufe of a wrong term (or, as he chufes to have it, a "barbarifm") for one of the re-agents employed; and a difcovery of my having found falts in this water, which he fays "*cannot poffibly exift together.*"

The fuppofed incorrectnefs of the term alluded to, I fhall not apologize for, having the authorities of Meffrs. Kirwan, Babington, and Aikin.

But, "has not that gentleman been rather unfortunate in his" other affertion? A flight acquaintance with the analyfes of mineral waters would have convinced him, that thefe falts *do* co-exift in various proportions in feveral of them:

For inftance, 1ft, He will find in the Philofophical Tranfactions, Vol. LXXXII. Part I. that Schmeisser obtained the fame variety of falts with myfelf from the *Kilburn Water*.

2dly. Dr. Garnet, on analyfing the fulphureous waters of Harrogate, found both muriat of lime and fulphat of magnefia, in the proportions of 13 grains of the former, and 10,5 of the latter in a gallon.*

3dly. Mr. Kirwan, in his excellent Effay on the analyfis of mineral waters, has the following obfervations.†

"I once thought the labour of analyfing mineral waters might be much abridged, by obferving, when certain fpecies of falts were difcovered in it, what other fpecies would be decompofed by that or thofe already difcovered, and confequently could not (I thought) be fuppofed to co-exift with them, and of courfe needed not be fought for. But I foon found, both by my own experiments and by the obfervations of the moft accurate analyfts, that when both fpecies of antagonift falts, if I may fo call them, are very far from the point of faturation in a given folution, they may co-exift in it. The requifite diftance from the point of faturation is various in various fpecies of falts; the obfervations I have made on it I fhall mention, after producing fome examples of the co-exiftence of thefe feemingly incompatible ingredients."

He

* Vid. his Treatife upon this Water, publifhed in 1794.

† Vid. the Chapter on "Incompatible Salts," p. 136.

He then proceeds to give examples, which are followed by obfervations in his ufual perfpicuous manner.

Three of the examples I fhall quote, becaufe they prove the co-exiftence of thofe falts, which this gentleman confidently afferts, "cannot *possibly* exift together."

4thly, "ACCUM found 1,04 of a grain of muriated lime and 13,8 of cryftallized Glauber (fulphat of foda) in a pound of water. 5 Crell Beytr. p. 465.

5thly, "CORNETTE found 6 grains of muriated lime and 6 of Glauber, to retain their tranfparency in one pound of water, Mem. Paris, 1778, p. 345. This indeed is not conclufive, for ftill there might have been a decompofition; but in the water of Salins, he proves that Glauber and muriated lime co-exift until heated. Ibid. p. 342.

6thly, BERGMAN found half a grain of muriated lime, and half a grain of Glauber, in a *kan* of the waters of Odin, in Upfal, and alfo 1,5 grains of aerated foda, (carbonat of foda.) 1 Berg. p. 157."

I truft the foregoing teftimonies are fufficient to prove the *possibility* of the co-exiftence of thefe falts; it is therefore unneceffary for me to "reconcile my analyfis with the facts and quotations" of your Correfpondent, in order to demonftrate that it is not "erroneous."

I am, &c.

*Hampstead, Sept.* 12, 1801. JOHN BLISS.

